# Delayed (21 Days) Post Stroke Treatment With RPh201, a Botany-Derived Compound, Improves Neurological Functional Recovery in a Rat Model of Embolic Stroke

**DOI:** 10.3389/fnins.2020.00813

**Published:** 2020-07-31

**Authors:** Chunyang Wang, Michael Chopp, Rui Huang, Chao Li, Yi Zhang, William Golembieski, Mei Lu, Zadik Hazan, Zheng Gang Zhang, Li Zhang

**Affiliations:** ^1^Department of Neurology, Henry Ford Hospital, Detroit, MI, United States; ^2^Department of Physics, Oakland University, Rochester, MI, United States; ^3^Department of Biostatistics and Research Epidemiology, Henry Ford Hospital, Detroit, MI, United States; ^4^Regenera Pharma, Ness Ziona, Israel

**Keywords:** stroke recovery, neurological functional, ischemic, axon, myelin

## Abstract

**Background:**

Despite the recent advances in the acute stroke care, treatment options for long-term disability are limited. RPh201 is a botany-derived bioactive compound that has been shown to exert beneficial effects in various experimental models of neural injury. The present study evaluated the effect of delayed RPh201 treatment on long term functional recovery after stroke.

**Methods:**

Adult male Wistar rats subjected to embolic middle cerebral artery occlusion (MCAO) were randomized into the following experimental groups (*n* = 20/group): (1) RPh201 treatment, and (2) Vehicle (cottonseed oil). RPh201 (20 μl) or Vehicle were subcutaneously administered twice a week for 16 consecutive weeks starting at 21 days after MCAO. An array of behavioral tests was performed up to120 days after MCAO.

**Results:**

Ischemic rats treated with RPh201 exhibited significant (*p* < 0.05) improvement of neurological function measured by adhesive removal test, foot-fault test, and modified neurological severity score at 90 and 120 days after MCAO. Immunohistochemistry analysis showed that RPh201 treatment robustly increased neurofilament heavy chain positive axons and myelin basic protein densities in the peri-infarct area by 61% and 31%, respectively, when compared to the Vehicle treatment, which were further confirmed by Western blot analysis. The RPh201 treatment did not reduce infarct volume.

**Conclusion:**

Our data demonstrated that RPh201 has a therapeutic effect on improvement of functional recovery in male ischemic rats even when the treatment was initiated 21 days post stroke. Enhanced axonal and myelination densities by RPh201 in ischemic brain may contribute to improved stroke recovery.

## Introduction

Stroke is a leading cause of disability worldwide (Lozano et al., 2012; Murray et al., 2012). Over the past decade, a substantial decrease of stroke mortality was achieved largely due to advancements of acute stroke management such as use of statins (Flint et al., 2012; Scheitz et al., 2016). However, as a substantial proportion of stroke survivors suffer from long-term disability that imposes an enormous medical and societal burden (Wolfe, 2000; Feigin et al., 2014), development of effective treatments to improve neurological function for stroke patients are of major importance.

RPh201, an extract of gum mastic, is being developed by Regenera Pharma for the treatment of various neurological diseases. Mastic and extracts of gum mastic have been extensively used as an herbal remedy and dietary supplement for many centuries (Lev and Amar, 2002). Accumulating evidence suggests that mastic gum extracts exert potent anti-inflammatory, anti-oxidative, anti-bacterial, and anti-cancer properties in various experimental diseases (He et al., 2006; Kaliora et al., 2007; Gholami et al., 2016). *In vitro* and *in vivo* animal toxicology studies performed using RPh201 for up to 39 weeks have revealed no genotoxic effects or safety concerns (Ramot et al., 2018a, b). Currently, clinical evaluations on the safety and therapeutic effects of RPh201 for patients with central nervous system (CNS) diseases are underway (Cummings et al., 2019; Rath et al., 2019). In healthy volunteers and in patients with previous non-arteritic anterior ischemic optic neuropathy (NAION), RPh201 was found to be safe and well tolerated after repeat subcutaneous injection (Hazan et al., 2019; Rath et al., 2019). A recent Phase 2 clinical trial shows that long-term RPh201 treatment improves visual function in patients with previous NAION, which highlights the therapeutic potential of RPh201 for CNS diseases (Rath et al., 2019). However, the therapeutic effects of RPh201 on stroke have not been explored. Thus, the present study was under taken to investigate whether post stroke RPh201 treatment improves neurological functional recovery in rats after embolic stroke.

## Materials and Methods

All experimental procedures were approved by the Institutional Animal Care and Use Committee of Henry Ford Hospital. All experiments and outcome assessments were performed by observers blinded to the treatments.

RPh201 is a 5% (w/w) formulation of the drug substance in National Formulary–grade cottonseed oil stabilized with butylated hydroxytoluene. Vehicle is National Formulary–grade cottonseed oil stabilized with butylated hydroxytoluene.

### Animal Model

Male Wistar rats (Charles River Laboratories) 2–3 months old and weighing 350–400 g (*n* = 40) were subjected to embolic middle cerebral artery occlusion (MCAO), as previously described (Zhang et al., 2001). Briefly, an arteriotomy was made on the external carotid artery (ECA) and a single clot (40 mm in length) obtained from a donor rat was placed at the origin of the MCA via an endovascular catheter. Three hours after MCAO, rats were evaluated using a 5 point Longa score (0 = no neurologic deficit, 1 = failure to extend left forepaw fully, 2 = circling to the left, 3 = falling to the left, and 4 = does not walk spontaneously) (Longa et al., 1989). Rats with a Longa score below 2 were excluded. Rats in the Sham group (*n* = 13) received the same surgical procedures but without clot placement.

### Experimental Design and Treatments

To examine the effect of RPh201 on brain remodeling during stroke recovery rather than on the neuroprotection in acute stroke, rats were randomly assigned to 3 groups according to a pre-generated randomization schema: (1) RPh201 treatment (*n* = 20), (2) Vehicle treatment (*n* = 20), and (3) Sham (*n* = 13). RPh201 is a 5% (w/w) formulation of the drug substance in Vehicle (National Formulary–grade cottonseed oil stabilized with butylated hydroxytoluene). RPh201 at a dose of 20 μl or the same volume of Vehicle were subcutaneously (SC) administered twice a week starting at 21 days after MCAO, a time point in which infarction is fully developed and brain remodeling occurs (Du et al., 1996; Garcia et al., 1997). The dose of RPh201 was chosen based on previously conducted studies in rat models of stroke and vascular dementia (unpublished data). The current dosage is 16–120 times less than the doses used in the toxicology study in rats and is approximately equivalent to the 20 mg of RPh201 used in clinical trials according to the published dose conversion methods between rat and human (Nair and Jacob, 2016; Ramot et al., 2018b; Rath et al., 2019). Rats were then divided into two cohorts for neurological function, histopathology, and immunochemistry evaluations at 2 weeks (35 days after MCAO, *n* = 5/group for ischemic rats, *n* = 4 for Sham rats) and 16 weeks (133 days after MCAO, *n* = 15/group for ischemic rats, *n* = 9 for Sham rats) after initiation of treatment. For labeling mitotic cells, bromodeoxyuridine (BrdU, 100 mg/kg, Sigma) was administered (i.p) daily for 7 consecutive days starting at 21 days after stroke or Sham operation.

### Neurological Outcome

Acute neurological outcome was evaluated at 3 h after MCAO using a 5 point Longa score (Longa et al., 1989; Zhang et al., 1997). Neurological outcome was also evaluated in all rats with modified neurological severity scores (mNSS), foot-fault test, and adhesive removal test performed at 1, 7, 14, 21, 28, 60, 90, and 120 days after MCAO or until the designated endpoints.

### mNSS

For a composites core of stroke induced impairments on motor, sensory, reflex, and balance ability, mNSS was graded on a scale of 0 (normal) to 18 (maximal deficit), as previously described (Chen et al., 2001).

### Adhesive Removal Test

Rats were tested for the responsiveness to forelimb somatosensory stimulation with the adhesive removal test. Briefly, 2 pieces of adhesive-backed paper (113.1 mm^2^) were used as bilateral tactile stimuli occupying the distal-radial region on the wrist of each forelimb for up to 28 days after stroke onset (Shen et al., 2007). Thereafter, half-sized stimuli (56.6 mm^2^) were used to increase the difficulty of the adhesive removal test starting 60 days post stroke. The mean time (seconds) required to remove the adhesive tab from the left forelimb was recorded with a cut off time at 120 s.

### Foot-Fault Test

Rats were tested for placement dysfunctions of forelimbs with the foot-fault test (Hernandez and Schallert, 1988). The total number of steps (movement of each forelimb) that the rat used to cross the grid and the total number of foot-faults (fall or slip between the wires) for the left forelimb were recorded. Data are presented as a percentage of left foot-faults.

### Infarct Volume Measurement

For rats enrolled into the 16 week treatment cohort, ischemic (*n* = 7 in Vehicle and *n* = 9 in RPh201 treatment group) and Sham rats (*n* = 5) were transcardially perfused with 0.9% saline and 4% paraformaldehyde 133 days after MCAO or Sham operation, respectively. Brains were fixed in 4% paraformaldehyde and embedded in paraffin. A series of coronal sections (6 μm) were cut for histology and immunohistochemistry analysis. Infarct volume was measured on Hematoxylin & Eosin stained coronal sections, as previously described (Zhang et al., 1997).

### Immunohistochemistry Analysis

Immunohistochemistry was performed on brain coronal sections within the MCA territory (bregma 0 to −1.4 mm) obtained from rats sacrificed 133 days after ischemia and Sham operation. The following antibodies were used: rat anti-Myelin basic protein (MBP, Millipore), chicken anti-Neurofilament heavy chain (NFH, ThermoFisher Scientific), rabbit anti-Synaptophysin (Abcam), for quantification of MBP, NFH, and Synaptophysin immunoreactivity. Immunoreactive areas were digitized throughout the peri-infarct area, as well as the corresponding contralateral homologous areas. The peri-infarct area was defined as a strip of cerebral tissue (300 μm in width) along the medial border of the infarct cavity (Ueno et al., 2012). Data are presented as the percentage of immunoreactive area at the peri-infarct region relative to the contralateral homologous region on the same section. Additional antibodies used for immunohistochemistry were: BrdU (Dako), Doublecortin (DCX, Abcam), Microtube associated protein 2 (MAP2, Aves Labs), Glial fibrillary acidic protein (GFAP, Dako), Ionized calcium binding adaptor molecule 1 (IBA1, Wako Chemicals), and CD-31 (R&D Systems). The density of BrdU-immunoreactive cells, the percentage DCX-immunoreactive area, and the percentage of BrdU and DCX double-labeled cells within the ipsilateral subventricular zone (SVZ) and peri-infarct area were measured. The number of IBA1 immunoreactive cells and the areas for MAP2 and GFAP throughout the peri-infarct area were measured. The density of CD-31 immunoreactive vessels and the percentage of BrdU and CD-31 double-labeled cells throughout the peri-infarct area was measured.

### Western Blot Analysis

Brain tissues were collected for rats enrolled into the 2 week treatment cohort at 35 days after MCAO (*n* = 5/Vehicle, *n* = 4/RPh201, and *n* = 4/Sham), and for rats enrolled into the 16 week treatment cohort at 133 days after MCAO (*n* = 5/Vehicle, *n* = 5/RPh201 treatment, and *n* = 4/Sham). Briefly, rats were decapitated under deep anesthesia. Brains were separated into left and right hemisphere and flash-frozen at −80°C for subsequent protein extraction. Total proteins were extracted for Western blots. The following primary antibodies were employed: rat anti-Myelin basic protein (MBP, Millipore), chicken anti-Neurofilament heavy chain (NFH, ThermoFisher Scientific), and rabbit anti-Synaptophysin (Abcam). The protein levels of MBP, NFH, and Synaptophysin were determined by Western blot analysis. Additional antibodies used for Western blot analysis were: Vascular endothelial growth factor receptor (VEGFR, Abcam), Intercellular adhesion molecule 1 (ICAM, Cell Signaling), Toll-like receptor-2 (TLR2, Abcam), and Toll-like receptor-4 (TLR4, Abcam).

### Statistical Analysis

Data were evaluated for normality, and ranked data or non-parametric Kruskal–Wallis test were used for data that were not normally distributed. For the safety evaluation, the log rank test/Kaplan-Meier was used to analyze mortality difference between RPh201 and Vehicle treated groups. The Global test using Generalize Estimating Equation (GEE, PROC GENMOD in SAS 9.4.) were implemented to test the treatment effect on the behavioral outcome measured from three behavioral tests at each time point (Zeger and Liang, 1986). Treatment effect was tested on individual outcome only if the Global test was significant at the 0.05 level. The one-way ANOVA was used to test the treatment effects on histology, immunohistochemistry, and Western blot measurements.

## Results

### Mortality and Safety

Prior to initiation of treatment, four rats (1 from RPh201 group and 3 from Vehicle group) died within 21 days after MCAO and these rats were excluded from further evaluation. During the treatment, one rat treated with RPh201 died at 7 days after initiation of treatment, while no rats died in the Vehicle group. Log rank test showed there were no significant (*p* = 0.79) survival differences between RPh201 and Vehicle groups. All ischemic rats lost body weight over the first week after MCAO, but gradually regained weight over time at a similar rate between the two groups. There was no treatment effect on body weight (*p* = 0.583) in the ischemic rats.

### RPh201 Treatment Improves Functional Recovery

**FIGURE 1:**
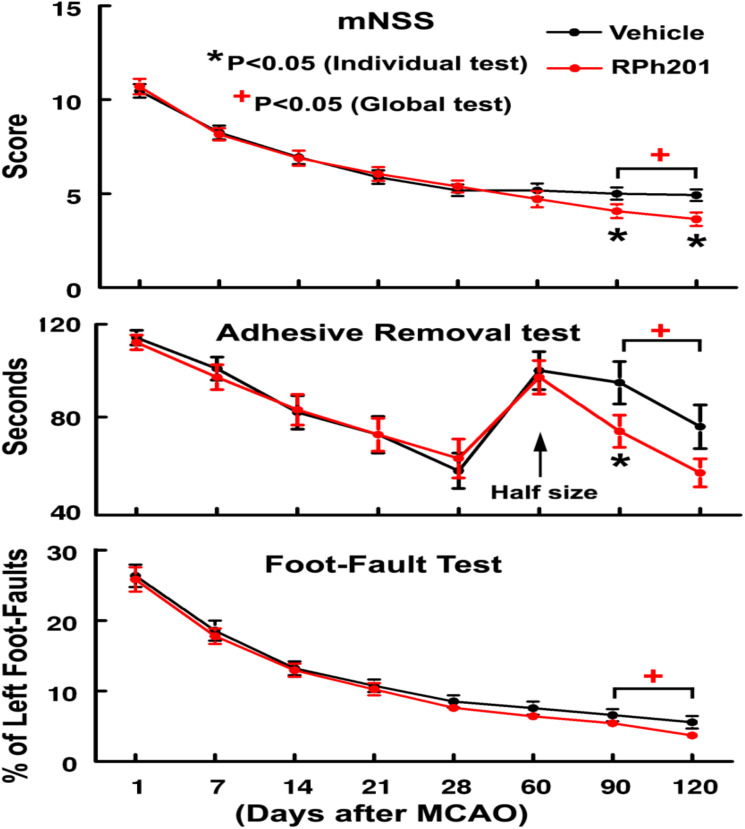
The effect of RPh201 on neurological outcome. Neurological outcome measured by mNSS, adhesive removal test, and foot-fault test in ischemic rats treated with RPh201 and Vehicle. Values are mean ± SE. *^, +^*p* < 0.05 compared with the Vehicle group.

The Global test revealed that Sham operated animals did not show any neurological deficits throughout the experiments; however, all ischemic rats exhibited neurological functional deficits measured by mNSS, adhesive removal test, and foot-fault test prior to the treatment (at 1, 7, 14, and 21 days after MCAO), which were comparable between RPh201 and Vehicle groups, indicating that the baseline neurological functions were balanced among groups. The RPh201 treatment significantly reduced neurological deficits starting from day 90 and persisting to day 120 after MCAO compared to the Vehicle treatment (Figure 1).

Histopathological analysis showed that there were no significant differences of infarct volume (*p* = 0.5) between the rats treated with RPh201 (30 ± 12.4%, *n* = 9) and Vehicle (25.9 ± 10.1%, *n* = 7). No ischemic lesion was observed in sham operated animals.

### RPh201 Treatment Increases Axonal and Myelin Densities After Stroke

**FIGURE 2:**
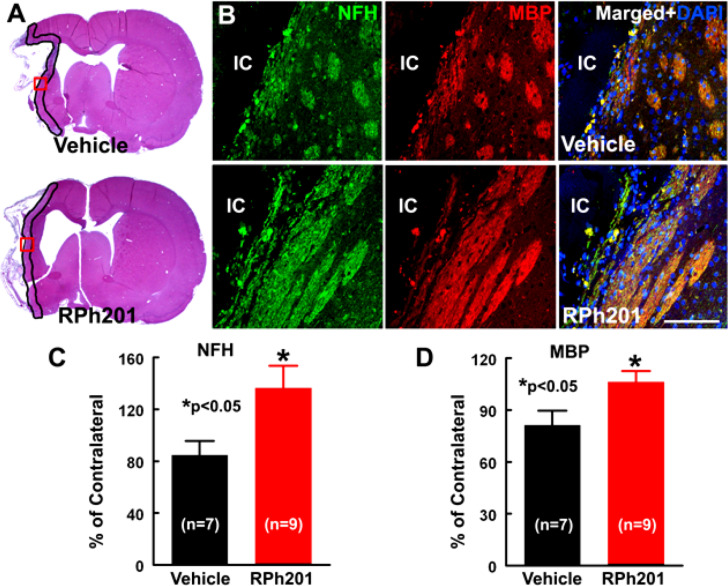
The effect of RPh201 on NFH and MBP immunoreactivity. Panels in **(A)** are the representative images of H&E stained coronal brain sections obtained from ischemic rats after 16 weeks of Vehicle and RPh201 treatments. The peri-infarct area in each section is outlined in black. Panels in **(B)** are the double immunofluorescent images of NFH (green) and MBP (red) immunoreactivity within the peri-infarct areas (boxed areas in **A**) acquired from adjacent coronal sections from **(A)**. Bar graphs are the quantitative data of NFH **(C)** and MBP **(D)** immunoreactive areas in the peri-infarct region. IC: ischemic core. Bar = 100 μm. Values are mean ± SE. *p < 0.05.

**FIGURE 3:**
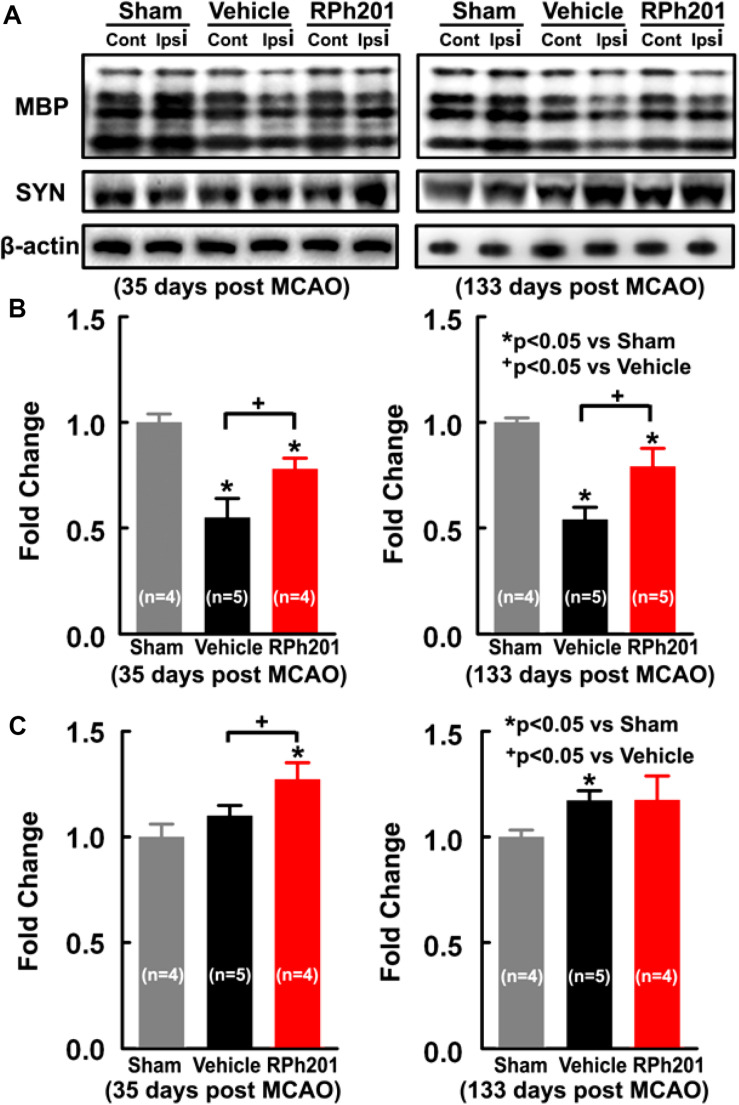
The effects of RPh201 on protein levels of MBP and synaptophysin. Representative Western blot images **(A)**, and quantitative date of MBP **(B)** and Synaptophysin **(C)** proteins in brain tissue after 2 and 16 weeks of RPh201 and Vehicle treatment.

Neuronal and myelin remodeling contributes to stroke recovery (Hermann and Chopp, 2012; Zhang et al., 2013; Zhang and Chopp, 2015; Jones, 2017). We thus, examined the effect of RPh201 on axons, dendrites, and myelin. Immunohistochemical staining analysis showed that the RPh201 treatment significantly increased the densities of NFH+ axons and dendrites and MBP+ myelin in the peri-infarct region 133 days after MCAO compared with the Vehicle treatment (Figure 2). However, the RPh201 treatment did not significantly affect synaptophysin+ synapse density in peri-infarct area (47 ± 5%) compared with the Vehicle (56 ± 4%). No statistically significant differences were detected in BrdU, DCX, MAP2, IBA1, GFAP, CD-31 immunoreactivities between RPh201 and Vehicle treatment groups (data not shown). Western blot analysis showed that the RPh201 treatment significantly increased MBP protein levels in the ipsilateral hemisphere compared with the Vehicle treatment at 35 and 133 days after MCAO (Figure 3). Moreover, RPh201 treatment significantly increased the ipsilateral synaptophysin levels at 35 days but not at 133 days after MCAO, compared with the Vehicle treatment. However, the ipsilateral NFH protein levels were not significantly different at 35 and 133 days after MCAO between RPh201 and Vehicle groups (*P* = 0.79, data not shown). In addition, no differences were detected in ipsilateral hemispheric VEGFR, ICAM1, TLR2, and TLR4 levels between RPh201 and Vehicle groups (data not shown).

## Discussion

Our data for the first time demonstrated that administration of RPh201 initiated 3 weeks after stroke onset improves neurological functions. The RPh201 increased neuronal and myelination remodeling likely contributes to improved long-term stroke recovery.

Following ischemic stroke, oxidative stress and the inflammatory response are key pathological mechanisms that escalate brain damage. Treatment strategies that inhibit oxidative stress and inflammatory response following stroke may confer neuroprotection. Thus, a neuroprotective effect of RPh201 can be speculated. However, the RPh201 treatment initiated 21 days after MCAO did not reduce infarction, which is expected because infarction matures during 7 and 14 days after MCAO (Du et al., 1996; Garcia et al., 1997). These data suggest that the delayed (21 days) RPh201 induced improvement of neurological function is unlikely to result from its neuroprotective effects, but is likely attributed to the effect of RPh201 on enhancing recovery.

Stroke stimulates coordinated white matter remodeling processes including remyelination of demyelinated axons, axonal reorganization/regeneration, and synaptic formations, all of which in concert, contribute to stroke recovery (Murphy and Corbett, 2009; Filous and Schwab, 2018). Stroke induced white matter remodeling/repair processes are heightened within first few weeks after stroke onset, a period during which most robust recovery occurs (Cramer, 2008; Li et al., 2009; Krakauer et al., 2012). For example, remyelination occurs in the peri-infarct region starting a few days after stroke (Mandai et al., 1997; Gregersen et al., 2001; Tanaka et al., 2003; Arai and Lo, 2009; Mciver et al., 2010), whereas axonal spouting is present within 2 weeks, and elevation of peri-infarct synaptogenesis persists for 2 months after stroke onset (Stroemer et al., 1995). However, these spontaneous remodeling processes are limited. The present study showed that the RPh201 treatment significantly increased axonal and myelination densities in peri-infarct regions assessed by immunohistochemistry analysis 133 days after MCAO. Additionally, Western blot analysis revealed that RPh201 treatment augmented myelination and synaptic plasticity, demonstrated by elevation of MBP and Synaptophysin proteins as early as 35 days after MCAO, respectively. However, Western blot analysis did not detect a significant increase in NFH protein levels following the RPh201 treatment, which likely reflects differences of the two methods used to analyze proteins. The Western blot analysis was performed on bulk proteins extracted from the entire hemisphere tissue, while immunohistochemistry was done at the cellular level. Thus, immunohistochemistry provides a more sensitive method to detect differences of NFH levels in the peri-infarct regions between the treatment and vehicle group. Nevertheless, the present study suggests that RPh201 amplifies ischemic brain remodeling. Importantly, increase of MBP and Synaptophysin proteins by RPh201 occurs prior to significant improvement of neurological function that became apparent at 90–120 days after MCAO, suggesting that RPh201 amplified axonal and myelination remodeling in ischemic brain contributes to improved functional outcome. The effect of RPh201 on synaptophysin protein appears transient in which a significant augmentation was detected at 35 days, but not 133 days after MCAO. Additional experiments are warranted to investigate how RPh201 affects synaptophysin.

There are several limitations in the present study. First, the effect of RPh201 on cognitive function was not evaluated. Clearly, stroke induces long-term sensorimotor and cognitive dysfunction, and white matter remodeling after stroke is critical for cognitive performance (Wang et al., 2016). Thus, additional validation studies are warranted. Secondly, only young male rats were used in the present study, whereas stroke is primarily occurs older population with sexually dimorphic (Rothwell et al., 2005; Manwani and Mccullough, 2011). It remains to be investigated whether the current findings are applicable to ischemic aged rats, female rats, and animals with comorbid conditions. Thirdly, the present study did not assess the effect of RPh201 on temporal and spatial profiles of white matter remodeling, oligodendrogenesis, and the related activation of neuroinflammatory/neurotrophic factors. Finally, RPh201 is composed of a mixture of various constituents, including masticadienonic acid and isomasticadienonic acid (Hazan et al., 2019). Currently, the leading active substances for RPh201 remain to be identified. Further work is required to identify the key components that are responsible for the therapeutic effect of RPh201 for stroke treatment. Although a full spectrum of potential beneficial effects of RPh201 for stroke warrants further investigation, the present study highlights that during the chronic recovery phase, ischemic brain remains receptive to RPh201 treatment which amplifies white matter remodeling and promotes long-term recovery.

In conclusion, our data demonstrated that delayed 21 days onset of treatment with RPh201 effectively improves long-term neurological function in rats after stroke. RPh201 is well tolerated in the clinical setting. With emerging recognition that the long-lasting brain remodeling capacities following CNS injuries contribute to functional recovery, our data suggest that RPh201 has potential to aid recovery of neurological function in stroke survivors.

## Data Availability Statement

All datasets presented in this study are included in the article/supplementary material.

## Ethics Statement

The animal study was reviewed and approved by the Institutional Animal Care and Use Committee of Henry Ford Hospital.

## Author Contributions

CW, RH, CL, YZ, and WG performed the experiments. LZ, MC, and ZZ organized the database and contributed to conception and design of the study. ML performed the statistical analysis. CW and LZ wrote the first draft of the manuscript. ZH wrote sections of the manuscript. All authors contributed to manuscript revision, read and approved the submitted version.

## Conflict of Interest

ZH was employed by Regenera Pharma. The remaining authors declare that the research was conducted in the absence of any commercial or financial relationships that could be construed as a potential conflict of interest.

## References

[B1] Arai K. Lo E. H. (2009). Experimental models for analysis of oligodendrocyte pathophysiology in stroke. *Exp. Transl. Stroke Med.* 1:6. 10.1186/2040-7378-1-6 PMC2820444 20150984

[B2] Chen J. Sanberg P. R. Li Y. Wang L. Lu M. Willing A. E. (2001). Intravenous administration of human umbilical cord blood reduces behavioral deficits after stroke in rats. *Stroke* 32 2682–2688. 10.1161/hs1101.098367 11692034

[B3] Cramer S. C. (2008). Repairing the human brain after stroke: I. Mechanisms of spontaneous recovery. *Ann. Neurol.* 63 672–687. 10.1002/ana.21393 18383072

[B4] Cummings J. Lee G. Ritter A. Sabbagh M. Zhong K. (2019). Alzheimer’s disease drug development pipeline: 2019. *Alzheimers Dement.* 5 272–293. 10.1016/j.trci.2019.05.008 PMC6617248 31334330

[B5] Du C. Hu R. Csernansky C. A. Hsu C. Y. Choi D. W. (1996). Very delayed infarction after mild focal cerebral ischemia: a role for apoptosis? *J. Cereb. Blood Flow Metab.* 16 195–201. 10.1097/00004647-199603000-00003 8594050

[B6] Feigin V. L. Forouzanfar M. H. Krishnamurthi R. Mensah G. A. Connor M. Bennett D. A. (2014). Global and regional burden of stroke during 1990-2010: findings from the Global Burden of Disease Study 2010. *Lancet* 383 245–254. 24449944 10.1016/s0140-6736(13)61953-4 PMC4181600

[B7] Filous A. R. Schwab J. M. (2018). Determinants of axon growth, plasticity, and regeneration in the context of spinal cord injury. *Am. J. Pathol.* 188 53–62. 10.1016/j.ajpath.2017.09.005 29030051 PMC7338909

[B8] Flint A. C. Kamel H. Navi B. B. Rao V. A. Faigeles B. S. Conell C. (2012). Statin use during ischemic stroke hospitalization is strongly associated with improved poststroke survival. *Stroke* 43 147–154. 10.1161/strokeaha.111.627729 22020026

[B9] Garcia J. H. Liu K. F. Ye Z. R. Gutierrez J. A. (1997). Incomplete infarct and delayed neuronal death after transient middle cerebral artery occlusion in rats. *Stroke* 28 2303–2309; discussion 2310. 9368580 10.1161/01.str.28.11.2303

[B10] Gholami M. Ghasemi-Niri S. F. Maqbool F. Baeeri M. Memariani Z. Pousti I. (2016). Experimental and Pathalogical study of Pistacia atlantica, butyrate, Lactobacillus casei and their combination on rat ulcerative colitis model. *Pathol. Res. Pract.* 212 500–508. 10.1016/j.prp.2016.02.024 26972417

[B11] Gregersen R. Christensen T. Lehrmann E. Diemer N. H. Finsen B. (2001). Focal cerebral ischemia induces increased myelin basic protein and growth-associated protein-43 gene transcription in peri-infarct areas in the rat brain. *Exp. Brain Res.* 138 384–392. 10.1007/s002210100715 11460777

[B12] Hazan Z. Adamsky K. Lucassen A. Levin L. A. (2019). A first-in-human phase 1 randomized single and multiple ascending dose study of Rph201 in healthy volunteers. *Clin. Pharmacol. Drug Dev.* 9 366–374. 10.1002/cpdd.720 31250992 PMC7187404

[B13] He M. L. Yuan H. Q. Jiang A. L. Gong A. Y. Chen W. W. Zhang P. J. (2006). Gum mastic inhibits the expression and function of the androgen receptor in prostate cancer cells. *Cancer* 106 2547–2555. 10.1002/cncr.21935 16691616

[B14] Hermann D. M. Chopp M. (2012). Promoting brain remodelling and plasticity for stroke recovery: therapeutic promise and potential pitfalls of clinical translation. *Lancet Neurol.* 11 369–380. 10.1016/s1474-4422(12)70039-x 22441198 PMC3964179

[B15] Hernandez T. D. Schallert T. (1988). Seizures and recovery from experimental brain damage. *Exp. Neurol.* 102 318–324. 10.1016/0014-4886(88)90226-9 3197789

[B16] Jones T. A. (2017). Motor compensation and its effects on neural reorganization after stroke. *Nat. Rev. Neurosci.* 18 267–280. 10.1038/nrn.2017.26 28331232 PMC6289262

[B17] Kaliora A. C. Stathopoulou M. G. Triantafillidis J. K. Dedoussis G. V. Andrikopoulos N. K. (2007). Alterations in the function of circulating mononuclear cells derived from patients with Crohn’s disease treated with mastic. *World J. Gastroenterol.* 13 6031–6036. 18023095 10.3748/wjg.v13.45.6031 PMC4250886

[B18] Krakauer J. W. Carmichael S. T. Corbett D. Wittenberg G. F. (2012). Getting neurorehabilitation right: what can be learned from animal models? *Neurorehabil. Neural Repair* 26 923–931. 10.1177/1545968312440745 22466792 PMC4554531

[B19] Lev E. Amar Z. (2002). Ethnopharmacological survey of traditional drugs sold in the Kingdom of Jordan. *J. Ethnopharmacol.* 82 131–145. 10.1016/s0378-8741(02)00182-4 12241988

[B20] Li L. Jiang Q. Ding G. Zhang L. Zhang Z. G. Li Q. (2009). Mri identification of white matter reorganization enhanced by erythropoietin treatment in a rat model of focal ischemia. *Stroke* 40 936–941. 10.1161/strokeaha.108.527713 19150870 PMC2730918

[B21] Longa E. Z. Weinstein P. R. Carlson S. Cummins R. (1989). Reversible middle cerebral artery occlusion without craniectomy in rats. *Stroke* 20 84–91. 10.1161/01.str.20.1.84 2643202

[B22] Lozano R. Naghavi M. Foreman K. Lim S. Shibuya K. Aboyans V. (2012). Global and regional mortality from 235 causes of death for 20 age groups in 1990 and 2010: a systematic analysis for the Global Burden of Disease Study 2010. *Lancet* 380 2095–2128. 23245604 10.1016/S0140-6736(12)61728-0 PMC10790329

[B23] Mandai K. Matsumoto M. Kitagawa K. Matsushita K. Ohtsuki T. Mabuchi T. (1997). Ischemic damage and subsequent proliferation of oligodendrocytes in focal cerebral ischemia. *Neuroscience* 77 849–861. 10.1016/s0306-4522(96)00517-9 9070757

[B24] Manwani B. Mccullough L. D. (2011). Sexual dimorphism in ischemic stroke: lessons from the laboratory. *Womens Health (Lond)* 7 319–339. 10.2217/whe.11.22 21612353 PMC3128473

[B25] Mciver S. R. Muccigrosso M. Gonzales E. R. Lee J. M. Roberts M. S. Sands M. S. (2010). Oligodendrocyte degeneration and recovery after focal cerebral ischemia. *Neuroscience* 169 1364–1375. 10.1016/j.neuroscience.2010.04.070 20621643 PMC3789594

[B26] Murphy T. H. Corbett D. (2009). Plasticity during stroke recovery: from synapse to behaviour. *Nat. Rev. Neurosci.* 10 861–872. 10.1038/nrn2735 19888284

[B27] Murray C. J. Vos T. Lozano R. Naghavi M. Flaxman A. D. Michaud C. (2012). Disability-adjusted life years (Dalys) for 291 diseases and injuries in 21 regions, 1990-2010: a systematic analysis for the Global Burden of Disease Study 2010. *Lancet* 380 2197–2223. 23245608 10.1016/S0140-6736(12)61689-4

[B28] Nair A. B. Jacob S. (2016). A simple practice guide for dose conversion between animals and human. *J. Basic Clin. Pharm.* 7 27–31. 27057123 10.4103/0976-0105.177703 PMC4804402

[B29] Ramot Y. Hazan Z. Lucassen A. Adamsky K. Ross V. Young N. (2018a). Toxicity and toxicokinetic study of subcutaneously administered Rph201 in minipigs. *Toxicol. Pathol.* 46 693–705. 10.1177/0192623318786428 30009686

[B30] Ramot Y. Hazan Z. Lucassen A. Adamsky K. Santhosh Kumar D. P. Vijayasarathi S. K. (2018b). Toxicity and toxicokinetic study of Rph201 in Sprague-Dawley rats. *Food Chem. Toxicol.* 112 168–177. 10.1016/j.fct.2017.12.036 29288761

[B31] Rath E. Z. Hazan Z. Adamsky K. Solomon A. Segal Z. I. Levin L. A. (2019). Randomized controlled phase 2a study of Rph201 in previous nonarteritic anterior ischemic optic neuropathy. *J. Neuroophthalmol.* 39 291–298. 10.1097/wno.0000000000000786 31430268 PMC6705418

[B32] Rothwell P. M. Coull A. J. Silver L. E. Fairhead J. F. Giles M. F. Lovelock C. E. (2005). Population-based study of event-rate, incidence, case fatality, and mortality for all acute vascular events in all arterial territories (Oxford Vascular Study). *Lancet* 366 1773–1783. 10.1016/s0140-6736(05)67702-1 16298214

[B33] Scheitz J. F. Macisaac R. L. Abdul-Rahim A. H. Siegerink B. Bath P. M. Endres M. (2016). Statins and risk of poststroke hemorrhagic complications. *Neurology* 86 1590–1596. 10.1212/wnl.0000000000002606 27016519 PMC4844236

[B34] Shen L. H. Li Y. Chen J. Cui Y. Zhang C. Kapke A. (2007). One-year follow-up after bone marrow stromal cell treatment in middle-aged female rats with stroke. *Stroke* 38 2150–2156. 10.1161/strokeaha.106.481218 17525391

[B35] Stroemer R. P. Kent T. A. Hulsebosch C. E. (1995). Neocortical neural sprouting, synaptogenesis, and behavioral recovery after neocortical infarction in rats. *Stroke* 26 2135–2144. 10.1161/01.str.26.11.2135 7482662

[B36] Tanaka K. Nogawa S. Suzuki S. Dembo T. Kosakai A. (2003). Upregulation of oligodendrocyte progenitor cells associated with restoration of mature oligodendrocytes and myelination in peri-infarct area in the rat brain. *Brain Res.* 989 172–179. 10.1016/s0006-8993(03)03317-1 14556938

[B37] Ueno Y. Chopp M. Zhang L. Buller B. Liu Z. Lehman N. L. (2012). Axonal outgrowth and dendritic plasticity in the cortical peri-infarct area after experimental stroke. *Stroke* 43 2221–2228. 10.1161/strokeaha.111.646224 22618383 PMC3404219

[B38] Wang Y. Liu G. Hong D. Chen F. Ji X. Cao G. (2016). White matter injury in ischemic stroke. *Prog. Neurobiol.* 141 45–60. 27090751 10.1016/j.pneurobio.2016.04.005 PMC5677601

[B39] Wolfe C. D. (2000). The impact of stroke. *Br. Med. Bull.* 56 275–286. 11092079 10.1258/0007142001903120

[B40] Zeger S. L. Liang K. Y. (1986). Longitudinal data analysis for discrete and continuous outcomes. *Biometrics* 42 121–130. 3719049

[B41] Zhang L. Zhang Z. G. Zhang R. L. Lu M. Adams J. Elliott P. J. (2001). Postischemic (6-Hour) treatment with recombinant human tissue plasminogen activator and proteasome inhibitor Ps-519 reduces infarction in a rat model of embolic focal cerebral ischemia. *Stroke* 32 2926–2931. 10.1161/hs1201.100207 11739997

[B42] Zhang R. Chopp M. Zhang Z. G. (2013). Oligodendrogenesis after cerebral ischemia. *Front. Cell Neurosci.* 7:201. 10.3389/fncel.2013.00201 24194700 PMC3810592

[B43] Zhang R. L. Chopp M. Zhang Z. G. Jiang Q. Ewing J. R. (1997). A rat model of focal embolic cerebral ischemia. *Brain Res.* 766 83–92. 10.1016/s0006-8993(97)00580-5 9359590

[B44] Zhang Z. G. Chopp M. (2015). Promoting brain remodeling to aid in stroke recovery. *Trends Mol. Med.* 21 543–548. 10.1016/j.molmed.2015.07.005 26278490 PMC4567429

